# On the Recent Advances in the Theory of Vision

**Published:** 1872-01

**Authors:** H. Helmboltz

**Affiliations:** The Royal Society of London


ART. III.—*On the Recent Advances in the Theory of Vision.* *By* H. HELMBOLTZ, *(of the Royal Society of London.)*

Translated from *Les Annales d'Oculistique*, BY F. W. ABBOTT, M. D.

The natural sciences and psychology, these two important branches of human knowledge, are found intermingled when we study the physiology of the senses. From this union result problems which interest equally the naturalist and the psychologist, and to the solution of which both must necessarily contribute. At first sight physiology appears to have to deal only with the material changes occurring in the bodily organs; the physiology of the senses ought, indeed, to consider in the first place nerves and their sensations, in so far as the latter are the result of irritation of the nerves. But when we examine the functions of organs, can we dispense with the study of the perceptions of external objects which are caused by nervous irritations? A sufficient motive to undertake this study is the fact that a perception often discloses a nervous irritation, or the modification of an irritation, which we would not otherwise have noticed. But the perceptions of external objects is unquestionably a conscious act of our faculty of representation; it is a *psychical* act. Moreover with the progress of the study of the perceptions we have seen a constant increase in the domain of these acts whose influence is perceived in the most elementary sensual perceptions. If these psychical acts have been studied but little hitherto it is because we have been accustomed to consider the perception of an external object as something furnished immediately by the sense and insusceptible of analysis.

It is scarcely necessary to refer here to the fundamental importance which these studies present relatively to almost all the other branches of science. Sensual perception is found, in fact, directly or indirectly at the base of every human cognizance, or conduces, at least occasionally, for the development of the diverse aptitudes of the human mind. It is the corner stone of all the relations between man and the external world, and the psychical functions which accompany these relations, even of the lowest and most simple order, are none the less important and worthy of interest. It is in this study that the art of experimenting, so improved by the study of the natural sciences, has been able to penetrate for the first time into the domain of the psychical functions. It is true that experiment can here be applied only to define the kind of sensual impressions which produce in us such or such a representation, but this research suffices us to draw numerous consequences as to the nature of the psychical processes which are found in connection, and it is in this sense that I wish to try to give an account of the results which have been given by recent researches concerning the physiological theory of vision.

Having just finished a complete work on Physiological Optics* I willingly profit by the occasion which is offered me to present in their connection (ensemble) the theoretical considerations of which I have just spoken. In the course of my work I have taken care to verify myself all the facts and all the experiments of any importance. We are, moreover, nearly at accord upon all the considerable experimental facts; there is scarcely any dispute except upon the value of certain individual differences which are presented in certain classes of perceptions. The remarkable progress which ophthalmology has made in the last few years has led many distinguished savans to study the physiology of vision, and in proportion as the quantity of facts has multiplied, it has become more easy to arrrange them and to form of them a satisfactory whole. Competent readers know, however, how much work is necessary to establish a great part of the most simple and apparently most incontestable facts.

In order to put this subject in a clear light it will be necessary, first, to define the physical functions of the eye considered as an

* Physiological Optics, by H. Helmboltz, Ninth volume of the General Encyclopedia of Physics, by G. Karstein, Leipsic, 1867.

optical instrument. We will next explain the physiological phenomena of irritation and transmission, of which the parts of the nervous system which pertain to the eye are the seat. In the last place we will examine the psychological question, how perceptions are the consequence of nervous irritations.

In the first part, which we cannot omit because it forms the basis of the rest, we shall doubtless find many things already generally known; it is necessary to repeat them, to be able to put the new facts in their proper place. This part of physics is also full of interest; upon it rests the extraordinary development which ophthalmology has taken in the past twenty years, a development which is perhaps without example in the history of medicine for its rapidity and its eminently scientific character. The philanthropist is not alone in rejoicing at its conquests, which prevent or cure so many ills in the presence of which we were so lately powerless; the friend of science has every reason to regard it with a proud eye. We must not deceive ourselves. It is not by groping or by chance that such progress has been made, but rather by an advance whose rigorous method is a guaranty of success. As of old, astronomy, by its example, gave to the physical sciences confidence in the true method, so at present, ophthalmology shows, in a striking manner, the progress which can be made in therapeutics by the application of proper methods of research, and by a knowledge of the causes of phenomena. It is not surprising that a field where a scientific spirit may promise itself such noble triumphs should have attracted capable workers; it is in great part due to their number that this development has been made with such surprising rapidity. I may be permitted to name for Germany, Holland, and England, Messers. Albert Von Graefe, of Berlin, Donders, of Utrecht, and Bowman, of London. Those who love Science for herself may cite here the profound verse of Schiller, "Let him who aspires to the favors of the Goddess seek not in her the woman." It is easy, in truth, to show that, in the subject which occupies us, the most important practical results have been the unexpected consequence of researches which a superficial spirit would have considered superfluous trifles; these studies, however, unfolded a succession of causes and effects, whose interest was at first appreciable only in a theoretical point of view.

### I.

### THE EYE CONSIDERED AS AN OPTICAL INSTRUMENT.

Among all the senses, vision has always been considered the most precious gift, and the most marvellous product of nature. Sung by poets, celebrated by orators, the eye has been praised by philosophers as showing the limits of what organic nature is capable of producing, and physicists have striven to imitate the organ which they considered the ideal of optical instruments. The enthusiastic admiration of which this organ has always been the object is easily appreciated, when we think of the services which it renders us. The eye reaches to the limits of space; with a marvellous rapidity, the most variously colored images paint upon it a thousand changing pictures, and the representations which it furnishes form a spectacle of which we can never become weary. It is by the eye alone that we become acquainted with the depths of space, and the innumerable and luminous worlds by which it is peopled; it is the eye alone which enables us to admire terrestrial landscapes, with their harmonious changes of light and shade, the plants so varied in form and color, the animals with their movements so full of grace and power. Next to the loss of life, the loss of sight appears to us the most terrible of all.

The precision and certainty with which vision enables us to appreciate the position, the distance, and the size of the objects which surround us, are of much greater importance than the esthetic joys for which we are indebted to the eye. In truth, the appreciations furnished by the vision guide man in all his movements; they are as indispensable to the seamstress whose fine needle finds its way through the midst of the most complicated labarynth of threads, as to the chamois hunter, whose life depends upon the just estimation of the chasm across which he must leap. On the other hand, the success of our movements, which are founded essentially upon the notions which vision gives us of the external world, serves as a perpetual test of the accuracy of these notions. If vision deceived us as to the position and distance of objects, we could not fail to perceive it immediately, because we should put our hand or foot upon the places where we thought we saw the objects which we wished to touch. This continual verification of the exactness of the teachings of vision, which is made by our movements, is what leads us to so full and entire confidence in the testimony of this sense, a confidence which no objection proposed by philosophy or psychology will be able to disturb in the slightest degree. Since this is so, is it astonishing that the eye has been considered as an optical instrument with which none of those which man constructs can vie in perfection? Is it not natural that we should have thought that we could explain, by the precision and the complication of its structure, the exactness and the variety of the functions with which it is endowed?

However, the exact study of ocular optics, such study as has been made the past ten years, has disclosed a remarkable deception in regard to the optical faculties of the eye; a deception which the criticism of facts has inflicted on more than one inconsiderate enthusiasm. But here, as in other analagous cases, it appears to me that a more complete understanding of the facts, instead of destroying enthusiasm, only increases it by justifying it. In truth, the eminent services which this little organ renders, cannot be contested; and if, in a certain sense we are obliged to abate our admiration, we find at once a sufficient compensation in other and unexpected marvels.

Be that as it may, we can imitate none of nature's works; if human art produces optical instruments attaining, as such, a higher degree of perfection than the eye, the instrument which nature has here fashioned is not less marvellous than any other of her works.

Considered as an optical instrument, the eye is a camera obscura Every one is acquainted with this apparatus, which photographers use in taking portraits or landscapes. A box, blackened inside, has, in front, certain lenses which refract the light and re-unite it in such a manner as to form, at the posterior part of the box, a picture of the objects situated before the instrument. At first, when he adjusts the focus, the artist receives the image on a ground glass. Upon this screen the image appears inverted; it presents a natural coloring and its contours are of an elegance and sharpness which defy the imitation of the most skillful artist. Then he replaces the ground glass with a prepared plate, upon which light produces certain chemical changes; stronger at the most illuminated points, weaker at the shaded points. These changes produce the daguerrean or photographic image.

The natural camera obscura of our eye is likewise blackened internally. The cuboidal box of wood is replaced by a rounded shell, the *sclerotic.* This tough, white membrane, a part of which is visible in the living, constitutes the white of the eye. The inner surface of the sclerotic is covered by the *choroid,* a thin membrane formed almost entirely of red, interlacing blood vessels and covered with black pigment. The empty space of the camera obscura of the photographer is filled in the eye with a transparent mass clear as water. At the anterior part of the eye, in place of the glass lenses of the camera obscura, the *cornea,* formed of a transparent cartilaginous substance and presenting a spherical convexity, is inserted into the sclerotic. Its position and curvature are unalterable, because it forms part of the external envelope of the eye. The photographer's lenses are not fixed; they are mounted in a sliding tube which the operator moves by means of a rack and pinion, so as to obtain clear images on the ground glass, whatever may be the distance of the object which he wishes to reproduce. The lenses must be moved farther from the ground glass, in proportion as the object to be represented is nearer to the apparatus. As it is necessary that the eye should receive upon its posterior surface clear images of objects at different distances, this organ must likewise contain a moveable part. This role is played by the *crystalline lens* which, situated a little back from the cornea, is almost entirely covered by the colored iris. Behind the circular aperture of the iris, which is called the *pupil,* the lens is uncovered, and the border of the pupil rests upon its anterior surface. Nevertheless, on account of its extreme transparency, the lens is not visible under ordinary conditions of illumination; we perceive habitually only the black fundus of the eye. The crystalline is a soft elastic body, very transparent, and convex upon both surfaces. At its circumference it is supported by a ligament, fluted like a lady's collar, the *Zonule of Zinn,* whose tension may be diminished by the *ciliary muscle,* a circular muscle whose insertion is fixed near the cornea. When the ciliary muscle contracts, the traction exerted upon the periphery of the lens being lessened, this lens can return upon itself by reason of its own elasticity, and the convexity of its surfaces increases. There results from this an increase of refracting power, and the eye becomes capable of tracing upon its posterior surface the image of nearer objects.

The normal eye in a state of repose sees distinctly distant objects; the tension of the ciliary muscle accommodates it for near objects. The mechanism of the accommodation, which I have just sketched, has been since the time of Kepler one of the greatest enigmas of opthalmology; the question was at the same time of great practical importance, on account of the numerous pathological imperfections of accommodation. There exists no other question in optics upon which so many contradictory theories have been built. The first step towards its solution is due to the English oculist Sanson, in whom we must recognize the merit of remarkable keenness of observation, on account of the discovery which he made, in the pupil, of feeble luminous reflections formed by the two surfaces of the lens. This phenomenon, insignificant in appearance and scarcely perceptible, was only visible in a dark place, with a bright light at one side, and with the observor standing in a particular place. These little images of Sanson were destined to shed great light upon an obscure part of science. They are in truth the first sign by which the presence of the lens in a living eye could be recognized. Sanson immediately employed these reflections in order to determine objectively whether the lens were in place in the diseased eye. Max Langenbeck first noticed the changes which these reflections undergo during accommodation. This discovery was utilized simultaneously by Cramer, of Utrecht, and by the author to determine exactly all the changes of the lens during accommodation. There is an instrument, called a heliometer, which astronomers employ to measure the small distances between the stars, in spite of the mobility of the heavens. I applied the principle of this heliometer to the measurement of the eye in motion. The ophthalmometer, constructed for this purpose, serves to measure, on the living eye, the curvature of the cornea, the curvature of the two surfaces of the lens and the distance between these two surfaces &c., with more exactness than we have been able to do it up to the present time, upon the dead eye, which permit us to measure the changes which the optical apparatus undergoes during accommodation.

From that time the question was settled from a physiological point of view. To these researches were added those of Oculists, especially Donders, upon the individual defects of accommodation, which had been called in general *myopia* and *presbyopia*. It was necessary to institute methods of determining with precision the power of accommodation of the least experienced and most ignorant patients. He discovered that under the names of *myopia* and *presbyopia* very different conditions had been confounded and this had rendered the choice of spectacles very uncertain. Certain very obstinate diseases, which had been considered nervous affections for lack of knowing how to account for them, were very easily explained by the existence of certain defects in the apparatus of accommodation, and yielded rapidly to the employment of suitable glasses. Donders has shown also that strabismus is usually the consequence of defects of accommodation, while Von Graefe had already demonstrated that myopia, neglected and becoming progressive, might cause distensions and hurtful deformities of the fundus oculi.

Thus science, by unexpectedly linking together the chain of causes and effects, has produced results as useful for the diseased, as interesting for physiologists.

It remains for us to speak of the screen which receives the optical image which is formed in the eye; this is the retina, the thin, membranous continuation of the optic nerve, which forms the innermost layer of the membranes which line the ocular globe. The optic nerve is a cylindrical bundle, formed of very fine nerve fibres, held together and protected by a very tough tendinous sheath. It penetrates the eye ball at its posterior part and a little to the nasal side. From this point of entrance the fibres of the optic nerve expand, radiating to all parts of the anterior surface of the retina. The extremities of these fibres have a singular formation; there are at first cells and nuclei analagous to those which are found in the gray matter of the brain; then, at the posterior surface of the retina, and forming the extremity of the nervous conduit, is a regular mosaic composed of fine cylindrical *rods*, and larger, flask-shaped formations, which are called *cones*. All these elements are crowded one against another and situated perpendicularly to the surface of the retina; the rods communicate with the smallest nerve fibres, the cones with the larger ones. Positive experiments show that this mosaic of rods and cones is that layer of the retina in which luminous sensibility resides, that is to say, this layer of the retina is the only one where the action of light can produce a nervous excitation.

The retina presents a remarkable spot situated a little to its temporal side and not exactly in the middle, as one might suppose. This place, by reason of its color, is called the *yellow spot* (*macula lutea*); the retina presents here a slight increase in thickness. A little depression (*fovea centralis*) occupies the centre of this spot; the membrane is very thin at this point because its composition is reduced to the elements indispensable to exact vision. The cones which constitute the regular and close mosaic, with which the little depression is lined, are here finer (1-100 of a millimetre in diameter) than in the other parts. The other and more or less imperfectly transparent retinal elements are here wanting, except the nuclei belonging to the cones, and the fibres necessary for the union of the cones with the rest of the nervous apparatus. The retinal vessels do not penetrate the *fovea centralis;* their terminations surround this dimple with a crown of very fine and delicate capillary loops.

The *fovea centralis* is of very great importance in vision, because this is the spot of the most exact perception of distances. It is here that the cones, the terminal sensitive elements, are pressed most closely together. We can assume that each one of them possesses its nerve fibre which, through the medium of the optic nerve, goes separately to the brain, there to give the impression which has been received, and that there the excitation of each one of these cones may occasion an isolated sensation.

The formation of optical images in a camera obscura rests upon the fact that all the rays which proceed from one luminous point and enter the apparatus undergo, in their passage through the lenses, such a refraction that they all unite again at one single point. Every converging lens produces the same effect. Let us allow the rays of the sun to fall on a converging lens, and hold a sheet of white paper at a suitable distance behind it. There are then two things to be noticed; in the first place, and we usually forget to mention this, the lens casts a shadow as though it were an opaque body, rather than composed of transparent glass; in the second place, in the centre of this shadow is seen a dazzling spot, it is the image of the sun. On account of the refraction in the glass, the light which would have illuminated the whole surface, if we had not interposed the lens which casts upon it its shadow, is reunited at the shining spot which is occupied by the little image of the sun. Instead of this luminary let us choose a luminous source *without appreciable dimensions*, such as the star Sirius; the light then unites in a *point* at the focus of the lens. At this place the paper is illuminated, so that the image of the star is given by an illuminated point of the paper. The light of a second fixed star, near the first, would concentrate to a second point of the paper; this point being illuminated would furnish therefore the image of the second star. If the light of this star is red, the point illuminated by this light would evidently appear red. If there are several stars in the neighborhood, each one would have its image on a different point of the paper, and each image would show the color of the corresponding star. Finally, if, in place of isolated luminous points like stars, we have a succession of shining points constituting a line or a luminous surface, to this object would still correspond a succession of luminous points upon the paper; here still, *all* the light coming from one single point is concentrated again in *one single point* of the paper, provided that this screen is at the proper distance. Each point of the paper is illuminated with the intensity and the color belonging to it, and receives none of the light emitted by the points of the object to which it does not correspond. Let us substitute for our screen a sensitive photographic plate; the prepared surface undergoes, throughout, changes from the effect of the light which reaches it. But, of the light which enters the instrument, each point of the sensitive surface receives *all* that and *none but* that which proceeds from the corresponding point of the object, and, consequently, is illuminated with an intensity proportional to that of the object. The value of the change produced upon the sensitive plate is, therefore, everywhere in proportion to the chemical intensity of the light of the corresponding parts of the object.

Now it is precisely the same with the eye; only the glass lenses are replaced by the cornea; and the crystalline, the screen or sensitive plate, by the retina. When a distinct optical image is formed upon the retina, each cone of this membrane receives exclusively the light which proceeds from a superficial element that is very small compared to the field of vision; the nervous fibre of each cone is excited by the light from this corresponding element only, and does not feel any other, while the light of the other points of the field of vision excites other fibres.

It is in this way that the light of each point of the field of vision produces an isolated sensation, that the equal or different intensity of the various points of the field of vision may be distinguished and separated in the sensation, and that these different impressions may be perceived separately.

When the eye is compared with any optical instrument whatever, we are struck first of all with its great superiority in extent of field of vision. Each eye embraces from right to left nearly two right angles (160° from right to left and 120° up and down) and both together embrace more than two right angles in the horizontal sense. The field of vision of our artificial instruments is generally very small, smaller, indeed, in proportion to the increase in the magnifying power. But it should be remarked that we exact of the instrument a perfect accuracy of the image throughout its whole extent, while the retinal image needs great distinctness only for the little space occupied by the yellow spot. The diameter of the *fovea* corresponds to about one degree in the field of vision, that is to the surface covered by the nail of the index finger when the arm is fully extended. In this small portion of the field of vision the perception is sufficiently exact to enable us to distinguish two points a minute apart, the sixtieth part of the size of the nail held as before stated. This distance corresponds to the breadth of a cone of the retina. All the other parts of the retinal image are seen with less distinctness and so much the more as we approach the limits of the retina. The image received by the eye is therefore comparable to a drawing whose centre is very finely finished while the rest is only roughly sketched. If we see distinctly at one time only a very small portion of that which surrounds us, as a compensation, an advantage which no optical instrument presents, the eye enables us to take in a very large expanse of space with sufficient clearness to prevent remarkable objects, and still less the changes which may occur in the field of vision, from escaping our notice. But if the objects are too small we cease to be able to distinguish them with the lateral parts of the retina. The sky lark which we hear singing "lost in the blue of space" (Goethe) is, in truth, lost for us so long as we do not succeed in bringing its image upon the *fovea;* but then we can perceive it.

[TO BE CONTINUED.]

